# Reconstruction and Regeneration of Composite Fingertip Injuries Using Acellular Bladder Matrix

**DOI:** 10.1016/j.jhsg.2024.11.014

**Published:** 2025-01-24

**Authors:** Usman Zareef, Anna Green, Caroline Moore, Hari Iyer, Brian Katt, Ajul Shah

**Affiliations:** ∗ Department of Orthopaedic Surgery, University of Maryland School of Medicine, Baltimore, MD; † Department of Orthopaedic Surgery, University of Pittsburgh Medical Center, Pittsburgh, PA; ‡ The Plastic Surgery Center, Institute for Advanced Reconstruction, Shrewsbury, NJ 07702; § Department of Orthopaedic Surgery, Rutgers Robert Wood Johnson Medical School, New Brunswick, NJ

**Keywords:** Amputation, Fingertip, Regeneration, Urinary Bladder Matrix

## Abstract

**Purpose:**

Traumatic fingertip amputations are one of the most encountered injuries in the emergency department requiring evaluation by a hand surgeon. Current management strategies vary widely. We describe the use of acellular urinary bladder matrix (UBM) in complex distal fingertip injuries involving bone, soft tissue, and nailbed.

**Methods:**

A prospective cohort of 47 patients with proximal fingertip amputations (36 Allen zone III and 15 Allen zone IV) underwent UBM application with resultant fingertip regeneration. Patients received the first application in the operating room. Subsequent applications were reapplied weekly in the clinic setting until fibrinous granulation tissue was observed (average 2.5 total applications). Patients performed daily dressing changes until regeneration was achieved.

**Results:**

The average time to regeneration was 8.4 weeks. The mean length deficit compared to the contralateral fingertip was 3.6 mm for zone 3 and 4.8 mm for zone 4 injuries. The static 2-point discrimination of the injured fingertip was 1.2 mm less sensitive compared to the contralateral uninjured finger in zone 3 injuries and 1.1 mm in the zone 4 cohort. Overall patient satisfaction measured on a 10-point Likert scale was 9.5. Seven complications were observed: 5 hook nail deformities, one bony exostosis requiring surgical excision, and one case of pyogenic granuloma.

**Conclusion:**

Application of UBM is a reliable way to promote composite regeneration of Allen III-IV fingertip injuries. Its use resulted in excellent patient satisfaction with minimal complications encountered. Urinary bladder matrix should be considered for use in the treatment of proximal fingertip amputations.

**Level of Evidence:**

Therapeutic IV.

Traumatic fingertip amputations are one of the most commonly encountered injuries in the emergency department that require hand surgery evaluation. Between 1997 and 2016, fingertip amputations in the United States had an incidence of 7.5/100,000 person-years.^1^ Clinical approaches to fingertip injuries vary based on the level of injury, structural involvement, and patient preference.^2^ Treatment modalities vary based on the level of injury, and different zones have been described in the distal fingertip.^3^, ^4^, ^5^ The Allen zone classification is the most commonly used of these and is depicted in Figure 1. Traditional approaches to management of fingertip amputations include healing by secondary intention with dressing changes (zone I, II), primary closure with excision of exposed bone, flap reconstructions (Zones III, IV), tripartite reconstruction, skin grafts, and replantation (Fig. 1).^6^, ^7^, ^8^, ^9^, ^10^, ^11^

**Figure 1:**
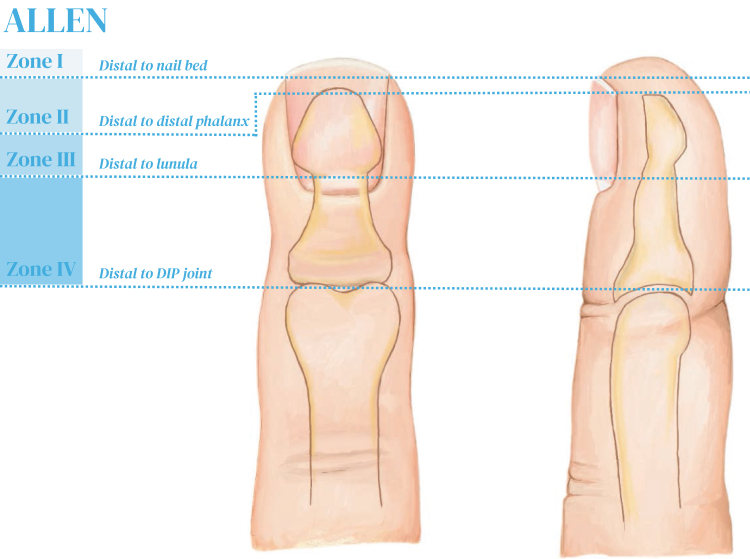
Depiction of Allen Zones I - IV.

In recent years, there has been growing interest in exploring alternative approaches to the management of fingertip amputations.^12^^,^^13^ One such approach involves the use of acellular urinary bladder matrix (UBM) powder for the treatment of proximal fingertip injuries. This novel application of UBM powder offers several advantages over traditional methods. Firstly, UBM provides a natural scaffold for cell growth and structural regeneration, promoting tissue healing and regeneration.^4^^,^^14^^,^^15^ Secondly, the use of UBM powder eliminates the need for complex flap reconstructions or replantation procedures, reducing surgical complexity and potential complications. Additionally, UBM powder can be applied easily to the wound site, simplifying the treatment process, and improving patient outcomes.^16^

The use of UBM has been well described for use in foot digit amputations, burn wounds, ulcers, and other reconstructive surgeries.^14^^,^^17^, ^18^, ^19^, ^20^ We implemented a novel approach to fingertip amputations at and around the lunula (Allen Zones III/IV) using acellular UBM. In this article, we present the details of this innovative approach and discuss its potential benefits in the management of proximal fingertip amputations.

## Methods

Institutional review board approval (No. IORG0007116) was obtained before investigation. The research was conducted in adherence with the Strengthening the Reporting of Observational Studies in Epidemiology guidelines. There was no external source of funding for this study. Sixty patients were referred by outside emergency facilities to three board-certified hand surgeons (B.K., H.I., A.S.). Patients were included if they were >18 years old, had an isolated Allen III-IV fingertip injury, and presented to the office after complete regeneration. Patients were excluded if their injuries were distal to zone III or if they were lost to follow-up. Forty-seven patients met inclusion criteria. All measurements and reporting of data are in adherence with the Standards for Reporting of Diagnostic Accuracy guidelines.

Patients were seen in the office on average 3.2 days (range, 0–15) after their injury, and subsequently in the operating room 5.6 days (range, 0–19) after initial consultation. In the operating room, each patient underwent surgical debridement followed by application of dry UBM. Adequate debridement was characterized as excision of nonviable tissue and development of a wound bed with high healing potential. In cases of bone exposure, the bone was not shortened to the level of soft tissue. Additionally, nails were not ablated in any case. For each patient, a 200 mg vial of UBM is used with a cost of $500 per vial. The finger then was covered with a Xeroform nonocclusive dressing directly on the wound, followed by Bacitracin ointment and a dry gauze wrap.

Patients were reassessed at weekly scheduled office visits, during which all dressings were removed and UBM was reapplied as needed until fibrinous granulation tissue was observed. If additional UBM was indicated, the dressings remained in place until the following visit. If no additional UBM was indicated, the wound was dressed with only Bacitracin, Xeroform, and gauze. One week after the final application of UBM, the patients were instructed to perform daily dressing changes, which consisted of Bacitracin directly and Xeroform applied to the finger until full epithelialization occurred. Finger range of motion exercises were encouraged after the initial postoperative visit to prevent stiffness of the treated finger.

Fingertip length and static 2-point discrimination (S2PD) were the primary outcomes measured. These measurements were performed in comparison with the same digit on the contralateral, uninjured hand. Fingertip length was measured from dorsal flexion crease to end of hyponychium. Measurements were completed after full regeneration during follow-up office visits. Secondary outcomes included number of UBM applications, time to regeneration, complications, and patient satisfaction, all of which were collected by the surgical care team.

## Results

In a final cohort of 51 digits, there were 36 Allen zone 3 and 15 zone 4 injuries. The average age of our study cohort was 45.8 years (range, 18–78) and included 40 males and 7 females. Injuries varied by digit and side: R1 (3), R2 (7), R3 (5), R4 (0), R5 (2), L1 (4), L2 (8), L3 (9), L4 (8), L5 (5), with multiple digits affected in two patients for a total of 51 digits. Table 1 demonstrates the demographics of the cohort by Allen zones. Two patients had multiple injured digits. Regeneration was categorized as full epithelialization and final measurements were made after regeneration was complete. Patients underwent a total of 2.6 ± 0.7 (range, 1–5) applications of UBM. On average, time to regeneration was 8.4 ± 1.9 (range, 4–12) weeks. Allen III injuries healed at a mean of 8.3 ± 2.0 weeks, and Allen IV injuries at 8.8 ± 1.8 weeks.

**Table 1:** Comparison of Baseline Demographics between Patient Cohorts with Allen zone III and IV Injuries

Allen zone	Demographic Variables					
	Age (y [SD])	Sex (% [M])	Hand Dominance (% [R])	UBM Applications (SD)	Days to Office (SD)	Days to OR from office (SD)
All (*n* = 51)	45.8 (17.1)	85.1%	87.50%	2.6 (.67)	3.2 (3.0)	5.6 (3.7)
III (*n* = 36)	47.1 (16.6)	80.6%	83.3%	2.5 (.61)	3.1 (2.9)	6.2 (3.6)
IV (*n* = 15)	44.2 (18.8)	92.9%	91.0%	2.9 (.70)	3.0 (3.4)	3.5 (2.6)

Data are presented as mean ±  SD, and all other variables are presented as *n* (%). OR, operating room.

**Figure 2:**
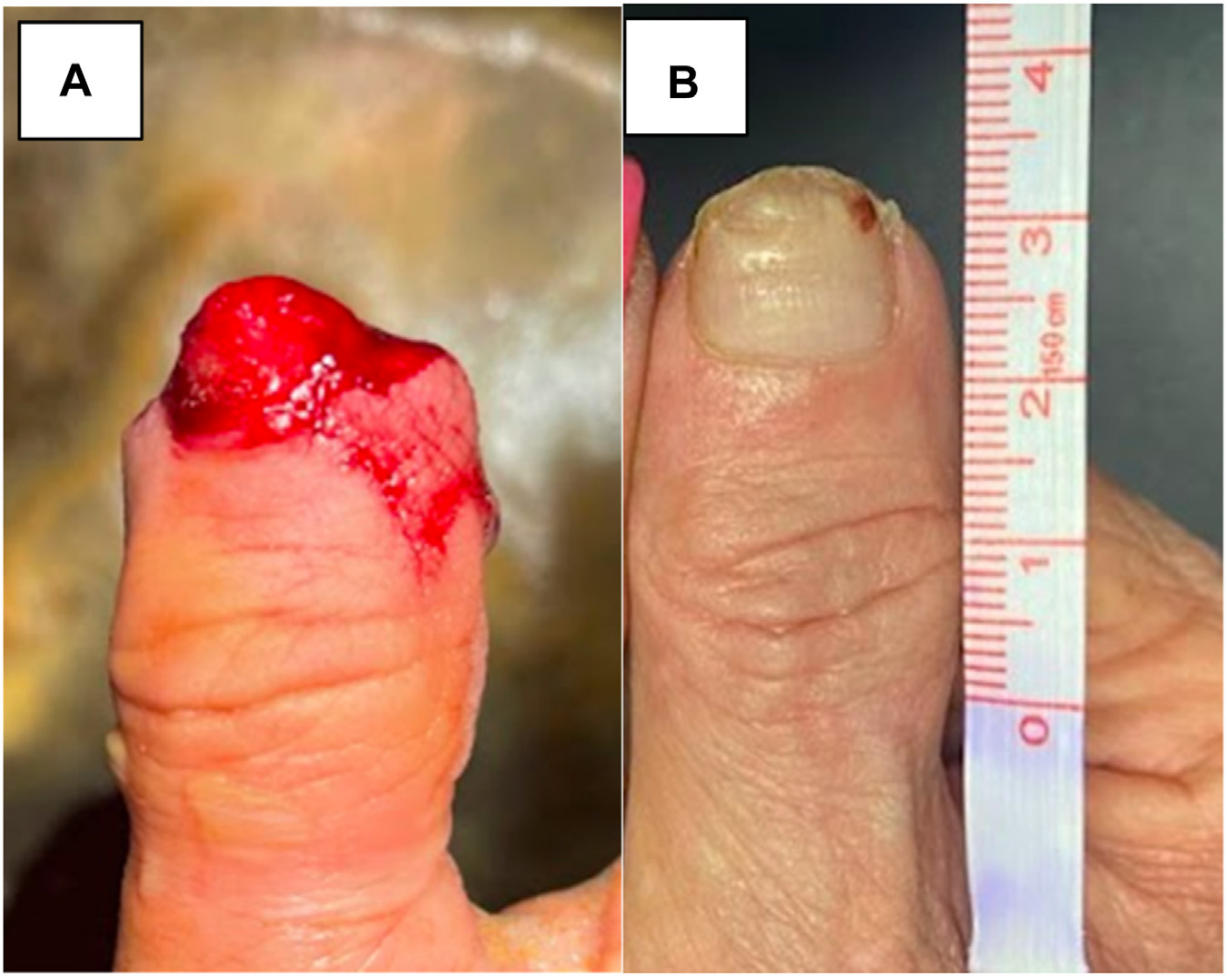
**A** Right thumb Allen 3 amputation just distal to the eponychial fold with exposed bone sustained by a 64-year-old woman smoker after getting crushed by a car door. The patient required 3 UBM applications and the injury healed in 6 weeks. **B** At 3-month follow-up, a near full fingertip with good contour was observed. The difference in length is measured best starting from the dorsal flexion crease.

**Figure 3:**
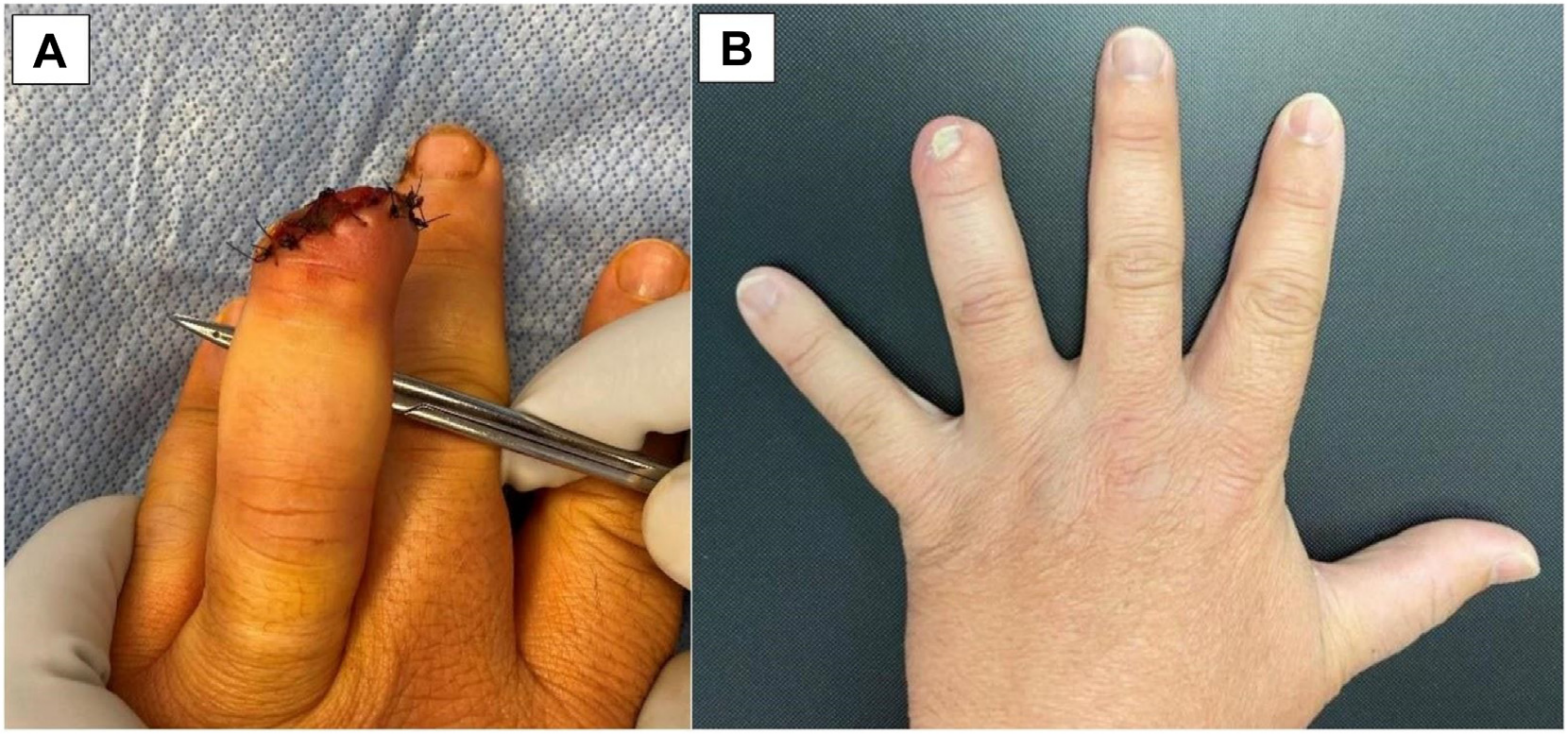
**A** Left ring Allen 4 injury proximal to the eponychial fold, which had been closed initially by the emergency department, in a 48-year-old nonsmoking man. The wound was opened, and the patient underwent three total matrix applications, which healed in 7 weeks. **B** 3-month follow-up shows partial nail regeneration.

**Figure 4:**
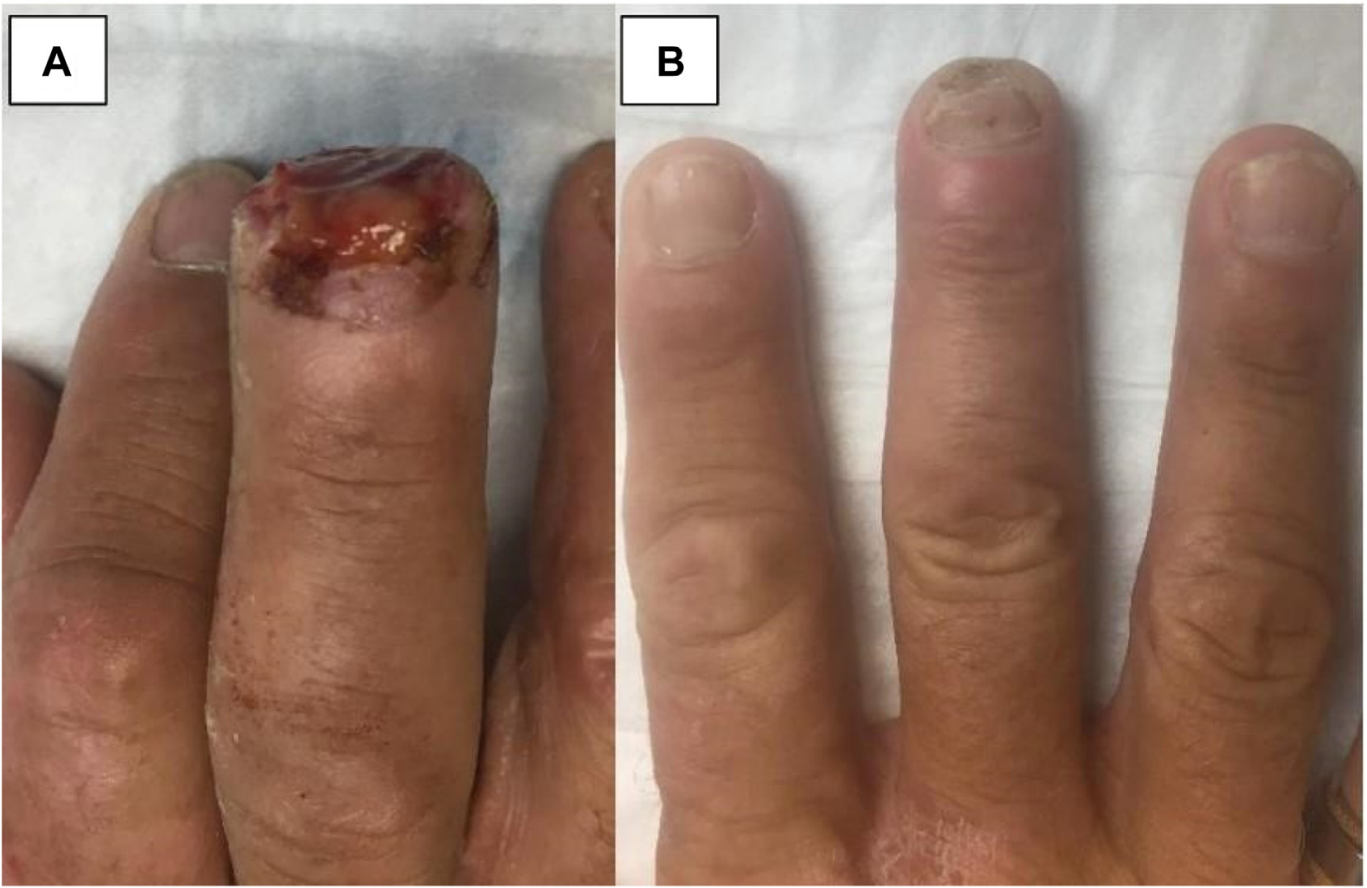
**A** Right middle finger Allen 4 injury immediately distal to the eponychial fold in a 47-year-old man who underwent 3 UBM applications. The healing process took 10 weeks. **B** Regeneration of the full fingertip with good contour and a slightly shorter nail is observed at 3 months.

**Figure 5:**
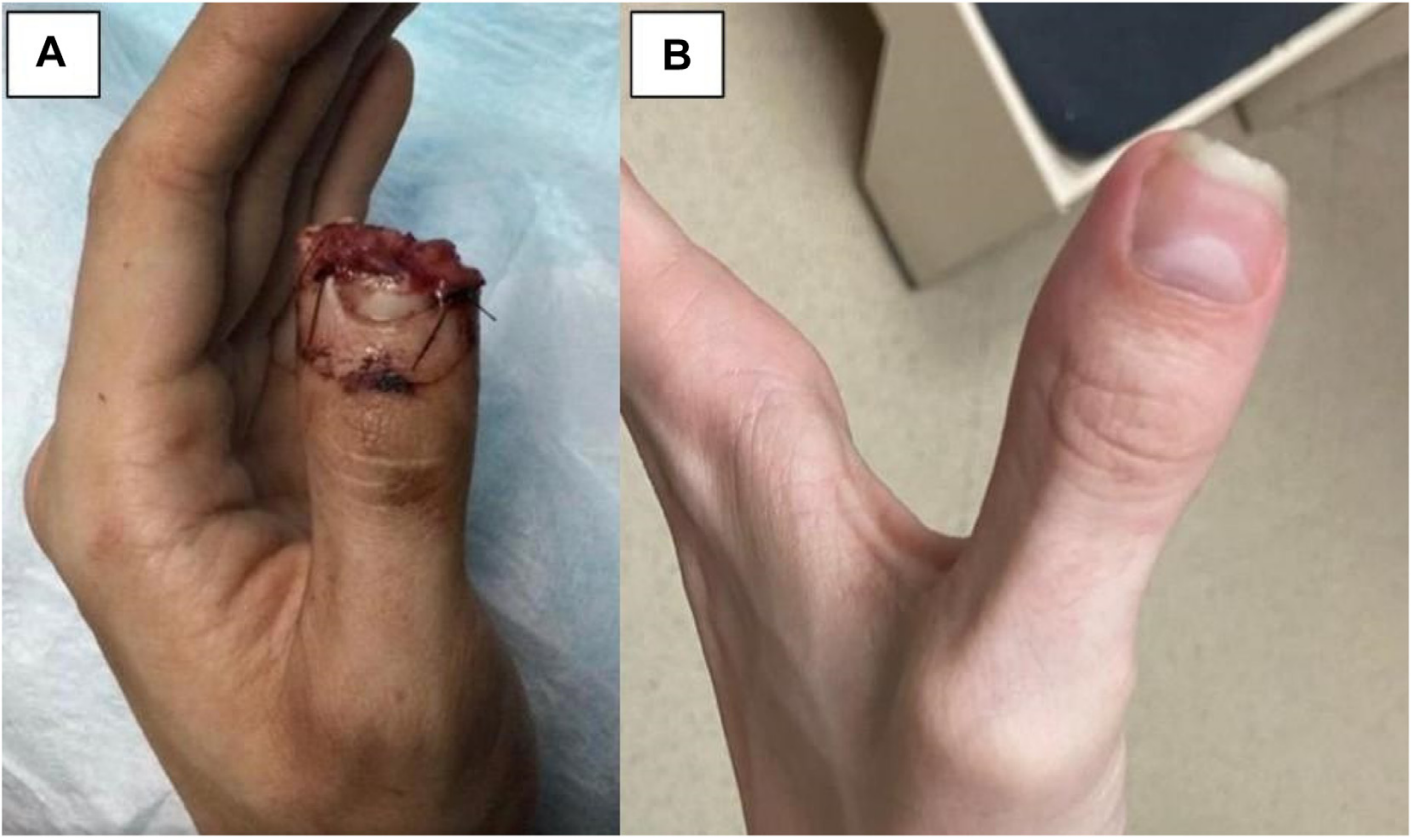
**A** 20-year-old man who sustained a composite Allen 3 amputation from a circular saw at the lunula of the left thumb. This patient underwent 3 UBM applications and the injury healed in 10 weeks. **B** Regeneration of the full thumb tip with nail and good contour was observed at 3 months.

Final measurements were taken after full regeneration was achieved. The mean length deficit compared to the contralateral fingertip was 4.0 ± 3.5 mm. Average static 2-point discrimination of the regenerated fingertip was 1.2 ± 0.98 mm less sensitive compared to the contralateral side. Average satisfaction on a scale from 1–10 (10 = most satisfied) was 9.5 ± 0.89 (range: 7.5-10). Average time to return to work was 4.5 ± 5.8 (range: 0-13) weeks. Table 2 depicts patient outcomes for the respective Allen zones. Select patient injuries and outcomes are depicted in Figure 2, Figure 3, Figure 4, Figure 5. Figure 2 shows right thumb Allen 3 amputation with fingertip regeneration at 3 months. Figure 3 shows left ring Allen 4 injury and partial nail regeneration at 3 months. Figure 4 shows right middle finger Allen 4 injury with fingertip regeneration at 3 months. Figure 5 shows a patient’s injury and outcome.

**Table 2:** Comparison of Clinical Outcomes Between Patient Cohorts with Allen zone III and IV Injuries

Allen Zone	Outcomes Measured					
	Complications, *n* (%)	Static 2PD Compared to Contralateral, mm (SD)	Length deficit Compared to Contralateral, mm (SD)	Time to Regeneration, Weeks (SD)	Time to Return to Work, Weeks (SD)	Patient Satisfaction, X/10 (SD)
All (*n* = 51)	7 (13.7%)	−1.2 (0.98)	4.0 (3.5)	8.4 (1.9)	4.5 (5.8)	9.5 (.89)
III (*n* = 36)	2 (5.5%)	−1.2 (0.96)	3.6 (2.7)	8.3 (2.0)	4.0 (5.9)	9.5 (.90)
IV (*n* = 15)	5 (35.7%)	−1.1 (1.06)	4.8 (4.9)	8.8 (1.8)	5.9 (5.8)	9.5 (.92)

Data are presented as mean ±  SD, and all other variables are presented as *n* (%).

There were seven complications in the cohort. Two hook nail deformities occurred in Allen III injuries (a left third digit in a 74-year-old diabetic man and a left second digit in a 23-year-old man). The patients did not want to pursue these further. Within the Allen IV cohort, there were five complications. There was a hook nail deformity in a 78-year-old man that sustained an Allen IV injury of his left small finger, one in a left second digit in a 70-year-old man, and another in the left fourth digit in a 54-year-old man. The patients did not want these deformities addressed. There was one bony exostosis in an Allen IV injury of the left ring finger that required surgical excision and healed with satisfactory aesthetic outcomes. Finally, one 28-year-old man experienced pyogenic granulosum after an Allen IV injury to his left long finger. This resolved with silver nitrate application three times over 3 weeks.

## Discussion

Management strategies vary widely for fingertip amputations, ranging from dressing changes to surgical reconstruction with the use of flaps and grafts.^2^ In this series, we describe the use of UBM in complex fingertip injuries involving bone, soft tissue, and nail bed with resultant composite regeneration and excellent outcomes.

Conservative management of fingertip injuries may lead to loss of motion, sensory deficits, and noticeable cosmetic deformity, all of which are more prevalent in proximal injuries. Boudard et al.^7^ studied treatment of fingertip amputees with occlusive dressings and reported nail dystrophy outcomes in all zone III injuries. Hoigne et al.^9^ studied treatment with semiocclusive dressing with scar-free healing of 90% of tissue in Ishikawa II/III (surrounding the lunula similar to Allen III-IV) injuries, but reported complications, such as a horned nail, hypersensitivity, and development of a neuroma. Van den Berg et al.^21^ reported that 90% of cases, when treated with either bone shortening or conservative approaches, had some form of nail deformity, with nearly 50% being parrot beak or hook nail deformities. Daily dressing changes can heal soft tissue pulp related injuries reliably, but are less reliable for more complex and proximal injuries.^7^^,^^9^^,^^17^^,^^21^ Excision of exposed bone with primary closure also is used commonly, which can leave patients with shortened and painful stumps.^6^

Traditional surgical options, such as flaps and skin grafts, risk donor site morbidity, necrosis, infection, finger stiffness, altered sensation, wound healing sequelae, fingertip instability and a lengthier rehabilitation time.^22^^,^^23^ in their review of fingertip injury treatments, Krauss et al.^6^ prefer a conservative approach with splinting and dressings rather than surgical treatment because of these associated risks.

The fingertips are essential for tactile sensation because of the high density of Pacini body receptors and branching of the digital nerves.^24^ Injury or partial amputation of the digit can have considerable effects on the normal ability for fine sensation. Normal S2PD is 3 to 4 mm.^25^ UBM offers the potential for improved fingertip sensation after partial amputation, with optimized restoration of sensation. The results of cross finger flaps have been shown to have S2PD of 6 mm or less, with the majority of injured fingers having within 2 mm of the contralateral uninjured finger sensitivity.^23^^,^^26^ Although cross finger flaps and other graft options offer acceptable outcomes, our sensate results are comparable and the UBM protocol is less invasive and does not risk donor site morbidity. Although semiocclusive dressing changes have shown return to normal sensation, these results were not segregated by zone of injury.^9^ Our results show excellent return of sensation at a mean loss of only 1.8 ± 0.98 mm compared to the contralateral uninjured digit. We hypothesize that this improved sensation is because of nerve regeneration that is influenced by the applied UBM matrix.

Composite regeneration of nail and soft tissues was observed in all cases in our cohort. This includes the injury that had been closed initially (Fig. 2), which would have led to a shortened fingertip and potential nail spicule growth. Our regeneration time was comparable to dressing change approaches reported in the literature for proximal composite injuries, with UBM healing in 8.1 weeks and nonocclusive dressing in 7 weeks.^21^ This cohort’s return to work time had a wide range of 0–13 weeks, which is likely predicated on several external factors, such as difference in profession, desire to return to work, and involvement of workers’ compensation. One concern regarding UBM is the increased cost because of operating room time and material. However, according to Frykberg et al.,^27^ UBM is of a significantly lower cost per patient than other common forms of graft materials, such as 1-stage Integra skin coverage and amniotic membrane, with a significant improvement in patient quality of life.^28^^,^^29^

Often, these patients will require emergency department consultation for hand surgery. The surgeons’ recommendations may vary from follow-up in the office to emergent bedside or operative procedures. The technique presented here simplifies and standardizes the evaluation and management of fingertip injuries, limiting the use of in-hospital resources and personnel. In their review on primary management of fingertip injuries in the emergency department, Hawken et al.^30^ endorse secondary management of amputations with dressing changes but acknowledge proximal injuries with bone involvement have increased risk of complications. In an acute setting, consideration of treatment options should include UBM, and patients can be counseled on potential regeneration and referred for hand surgeon follow-up and UBM application. Management in the acute setting traditionally has been focused on primary closure with more proximal amputation, also described as shortening and closing, of proximal injuries involving bone.^12^^,^^31^ Benefits of this procedure include faster return to work time. However, shortcomings reported include loss of additional digit length, and cold intolerance which may impact functionality.^12^

Limitations of this study include loss to follow-up, dependence on patient compliance with treatment, and the potential added cost of the UBM. UBM has been shown to delay complete wound healing and requires increased attention during the healing process but is considered inert because of its acellular nature and inflammatory reactions are uncommon.^32^^,^^33^ Patient-reported outcomes are subjective and are at risk for bias. It is important to note that the patients who did not present for final evaluation likely had an acceptable outcome and were not interested in further evaluation. This loss of follow-up would alter our complication rate.

We conclude that UBM application is a simple and reliable way to promote composite regeneration of Allen III-IV fingertip injuries with a low risk of nail deformity or surgical complications. Future directions should include a randomized control trial comparing UBM to dressing changes alone.

## Conflicts of Interest

No benefits in any form have been received or will be received related directly to this article.”
